# Energetics of proton release on the first oxidation step in the water-oxidizing enzyme

**DOI:** 10.1038/ncomms9488

**Published:** 2015-10-07

**Authors:** Keisuke Saito, A. William Rutherford, Hiroshi Ishikita

**Affiliations:** 1 Research Center for Advanced Science and Technology, The University of Tokyo, 4-6-1 Komaba, Meguro-ku, Tokyo 153-8904, Japan; 2 Department of Applied Chemistry, The University of Tokyo, 7-3-1 Hongo, Bunkyo-ku, Tokyo 113-8654, Japan; 3 Japan Science and Technology Agency (JST), PRESTO, 4-1-8 Honcho Kawaguchi, Saitama 332-0012, Japan; 4 Department of Life Sciences, Sir Ernst Chain Building, Imperial College London, London SW7 2AZ, UK

## Abstract

In photosystem II (PSII), the Mn_4_CaO_5_ cluster catalyses the water splitting reaction. The crystal structure of PSII shows the presence of a hydrogen-bonded water molecule directly linked to O4. Here we show the detailed properties of the H-bonds associated with the Mn_4_CaO_5_ cluster using a quantum mechanical/molecular mechanical approach. When O4 is taken as a μ-hydroxo bridge acting as a hydrogen-bond donor to water539 (W539), the S_0_ redox state best describes the unusually short O4–O_W539_ distance (2.5 Å) seen in the crystal structure. We find that in S_1_, O4 easily releases the proton into a chain of eight strongly hydrogen-bonded water molecules. The corresponding hydrogen-bond network is absent for O5 in S_1_. The present study suggests that the O4-water chain could facilitate the initial deprotonation event in PSII. This unexpected insight is likely to be of real relevance to mechanistic models for water oxidation.

## Abstract (web summary)

The availability of crystal structures of photosystem II opens up the possibility of gaining insights into its mechanism. Here, the authors use a computational approach and propose a deprotonation event at O4 followed by long-range proton-transfer along a chain of strongly bonded water molecules.

## Graphical abstract

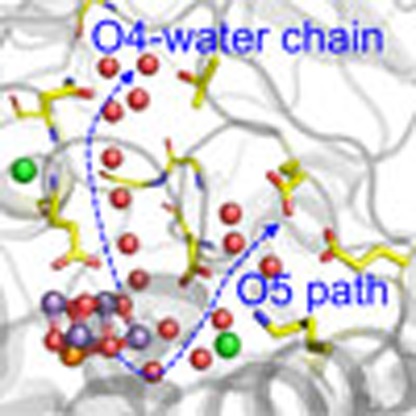

The core of the photosystem II (PSII) reaction center is composed of D1/D2, a heterodimer of protein subunits that contains the cofactors involved in photochemical charge separation, quinone reduction and water oxidation. In PSII, the Mn_4_CaO_5_ cluster catalyses the water splitting reaction: 2H_2_O→O_2_+4H^+^+4e^–^ (reviewed in refs 1, 2). The release of protons has been observed in response to changes in the oxidation state (the S_n_ state, where the subscript represents the number of oxidation steps accumulated) of the oxygen-evolving complex and occurs with a typical stoichiometry of 1:0:1:2 for the S_0_→S_1_→S_2_→S_3_→S_0_ transitions, respectively (for example, refs 3, 4). Although the relevant pathway for proton transfer (PT) in each S-state transition is not yet clear, PT may proceed via different pathways in the PSII protein, depending on the S-state transition^5^,^6^,^7^. Candidates for the relevant PT pathways have been reviewed recently^7^,^8^,^9^,^10^,^11^,^12^ and site-directed mutagenesis studies are testing the various possibilities^13^.

The majority view in the current literature is that the best resolved X-ray crystal structure (1.9 Å) of the Mn_4_CaO_5_ cluster^14^ represents an over-reduced form due to its reduction by the X-ray beam (for example, ref. 1 but see also for example, ref. 15 for an alternative explanation). This was suggested to explain the elongation of Mn–Mn and Mn–O distances compared with those obtained from extended X-ray absorption fine structure (EXAFS; for example, refs 16, 17, 18, 19, 20, 21). Detailed oxidation states of the 1.9-Å crystal structure are discussed in recent theoretical studies (see ref. 22 and references therein). Electron spin echo envelope modulation (ESSEM) and electron-nuclear double resonance (ENDOR) studies have suggested that all of the μ-oxo bridges of the Mn_4_CaO_5_ cluster are deprotonated in the S_2_ state, and that the water molecules, W1 and W2, bound to Mn4 are H_2_O or OH^–^ (refs 23, 24). Because proton release is not observed in the S_1_–S_2_ transition, the ESEEM and ENDOR data thus imply that the μ-oxo bridges of the Mn_4_CaO_5_ cluster are already deprotonated in S_1_ (refs 16, 19, 20, 21, 25). The 1.9-Å structure revealed that two water molecules, W1 and W2, were ligands to the Mn4 atom of the Mn_4_CaO_5_ cluster, and two waters, W3 and W4, were ligands to the Ca atom^14^. W3 has been proposed as a candidate deprotonation site because it is one of the closest water molecules to the redox active TyrZ^14^ and because the W3–Sr bond in the Mn_4_SrO_5_ cluster was specifically longer compared to that in the Mn_4_CaO_5_ cluster^26^. All of these bound water molecules are candidates not only as potential substrates for water oxidation (for example, refs 14, 27, 28, 29, 30, 31, 32) but also as ionizable groups that could undergo deprotonation during the enzyme cycle in which positive charge equivalents are accumulated^21^.

In recent experimental (for example, ref. 33) and theoretical studies (for example, refs 34, 35) it has been proposed that the proton released on the S_0_–S_1_ transition involves hydroxyl form of O5, an oxygen atom that occupies one of the corners of the cubane, linking the Ca, Mn3 and Mn1 and connecting the cubane to Mn4. Recently however, in the radiation-damage-free PSII crystal structure obtained using the X-ray free electron laser, which is assumed to be in the S_1_ state, Suga *et al*.^36^ proposed that O5 is a hydroxide ion in S_1_. This was based on the observation of significantly long distances between O5 and the adjacent Mn ions^36^. This would be conflict with the already deprotonated μ-oxo bridges in S_1_ suggested from the ESEEM and ENDOR data^19^,^20^,^21^. Recent report comparing published quantum mechanical/molecular mechanical (QM/MM) data and EXAFS data^37^ with the free electron laser structure^36^ suggested that the crystals used for this study could contain significant amount of the reduced S_0_ state^38^. Notably, the activation energy is specifically low in the S_0_–S_1_ transition, and can be rationalized as a rate-limiting electron transfer followed by a deprotonation step^21^.

The 1.9-Å structure shows the presence of a chain of strongly H-bonded eight-water molecules (O4-water chain) directly linked to O4 (linking Mn4 and Mn3 in the Mn_3_CaO_4_-cubane), whereas O5 has no direct-H-bond partner^14^. As far as we are aware, none of the QM/MM studies have considered the O4-water chain explicitly. In the 1.9-Å structure, the water molecule W539 (B-factor: monomer A=23.9, monomer B=25.2) is situated near the O4 atom of the Mn_4_CaO_5_ cluster^14^. Remarkably, the H-bond between O4 and W539 (O4–O_W539_) is unusually short (∼2.5 Å) in comparison with typical O–O distances of ∼2.8 Å for standard (asymmetric) H-bonds in H_2_O (refs 39, 40), even if we consider the uncertainty of ∼0.16 Å in the measured distances within the 1.9-Å structure^14^. Notably, ‘single-well H-bonds’ are very short and typically have O–O distances of 2.4–2.5 Å (refs 41, 42). This results in an essentially barrier-less potential between the H-bond donor and acceptor moieties. The appearance of single-well H-bonds in protein environments is often associated with PT events^43^,^44^,^45^.

Here we look for short H-bonds in the environment of the Mn_4_CaO_5_ complex and investigate their significance in energy terms by adopting a large-scale QM/MM approach based on the crystal structure analysed at 1.9 Å resolution^14^.

## Results

### A short H-bond between O4 and W539

**Figure 1:**
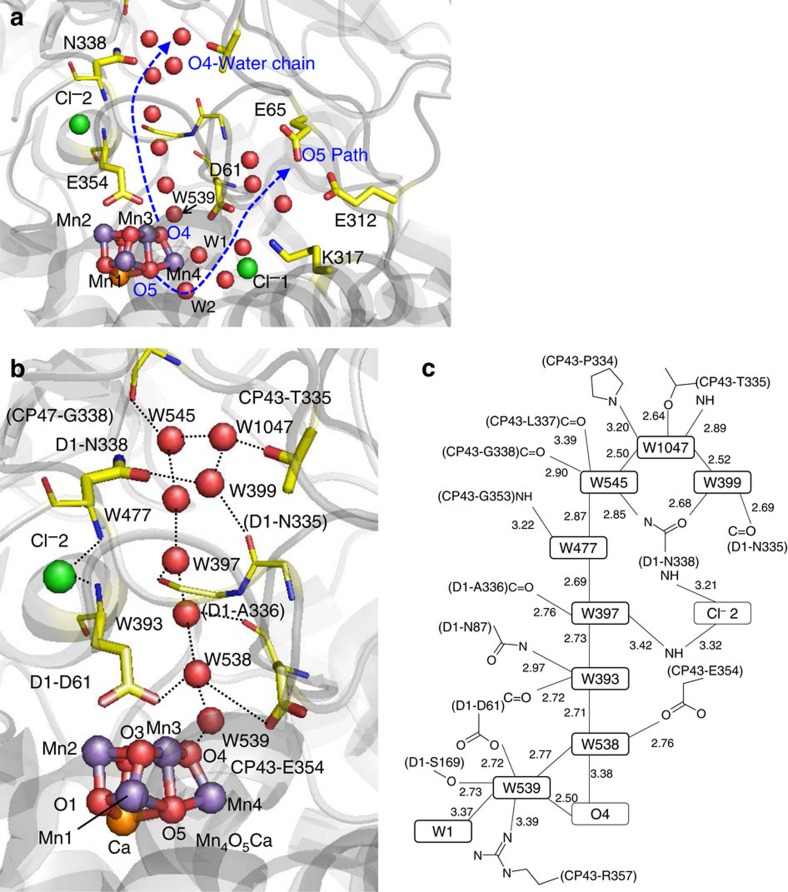
H-bond networks near the Mn_4_CaO_5_ cluster. (**a**) Overview. The O4-water chain is the H-bond network directly linked to the Mn_4_CaO_5_ cluster (O4–O_W539_=2.50 Å). The O5 path is not directly H-bonded to the Mn_4_CaO_5_ cluster (O5–O_W2_=3.08 Å). (**b**) The water chain linked to O4 of the Mn_4_CaO_5_ cluster in the 1.9-Å structure^14^. Water molecules (red), Cl^−^2 (green) and Mn_4_CaO_5_ (purple, orange, and red for Mn, Ca, and O atoms, respectively) are depicted as balls. H-bonds or ionic interactions are represented by dotted lines. (**c**) H-bond distances in the water chain (Å).

The O4–O_W539_ bond is unusually short in the original 1.9-Å structure (2.50 Å, Fig. 1)^14^. When an H-bond is assumed for the O4–O_W539_ bond, two H-bond patterns are possible. Case 1: the H atom is from O4, an OH^–^ moiety, so that the bond can be written O4–H…O_W539_. Case 2: the H atom is from the water, W539, so that O4 is O^2–^ and the bond can be written as O4…H–O_W539_ (Supplementary Fig. 1). Using these two cases, we investigated whether formation of the short H-bond between O4 and W539 is energetically possible in the S_–1_ to S_1_ states of the high oxidation state model. In the low oxidation state model these Mn valence states correspond to the *S*_*1*_–*S*_*3*_ states^15^ and this option was also investigated (Table 1), however in the present study, if not otherwise specified, the S-states given refer to the high oxidation state model. For discussions of the Mn oxidation state of the low oxidation state model^46^, see Supplementary Discussion.

**Figure 2:**
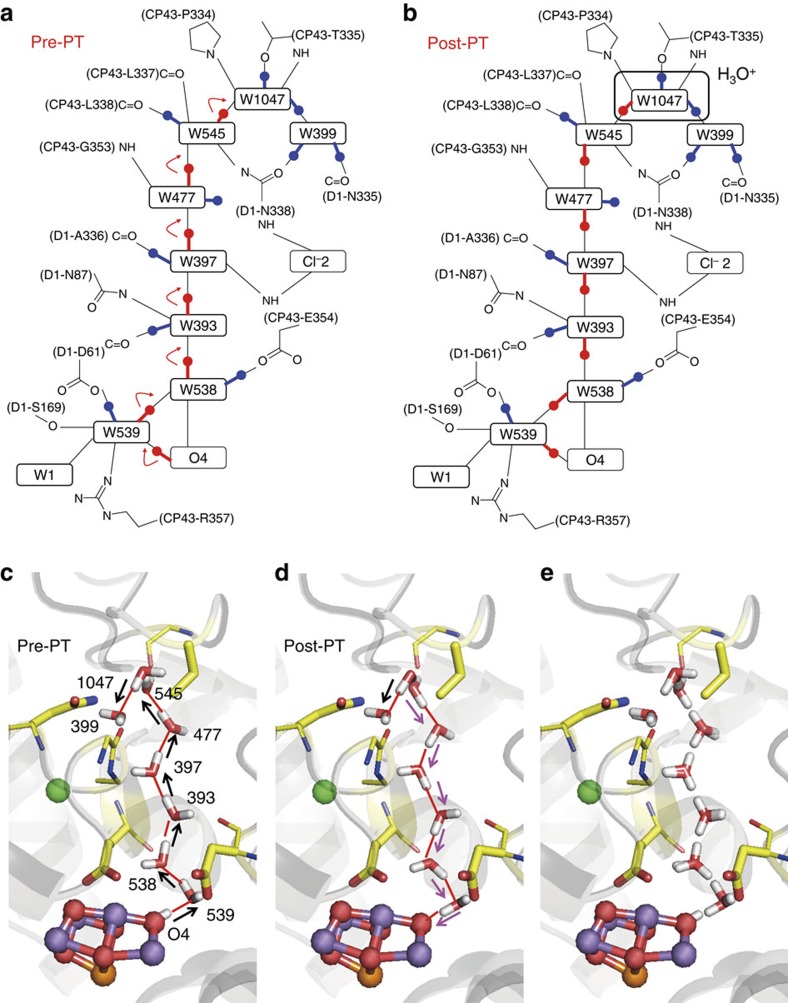
H-bond pattern change of the entire water chain induced by a release of a proton from O4. (**a**,**b**) The two H-bond patterns (pre- and post-PT) possess the same net charge and the same number of H atoms. Altered and unaltered H-bonds are indicated by red and blue lines and balls, respectively. (**c**) QM/MM optimized geometries in the pre-PT, (**d**) post-PT and (**e**) both conformations. Initial donor-to-acceptor orientations are indicated by black arrows, whereas altered donor-to-acceptor orientations in the post-PT conformation are indicated by magenta arrows.

**Figure 3:**
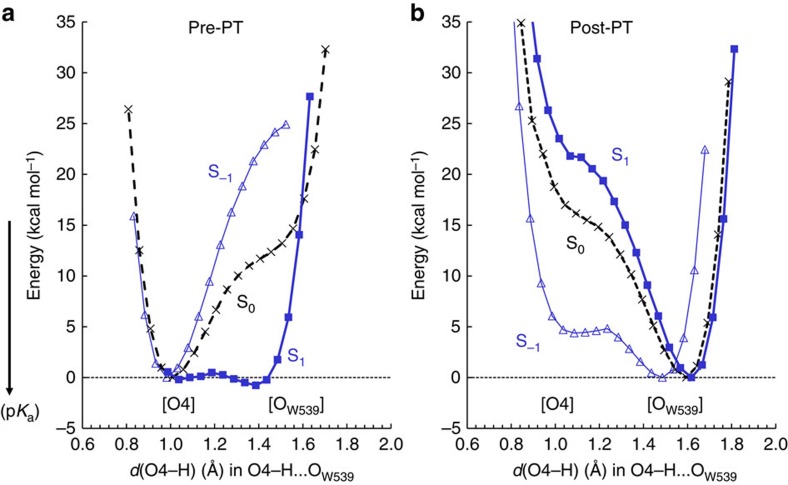
The energy profiles along the PT coordinate for the O4–O_W539_ bond. (**a**) pre-PT and (**b**) post-PT in S_1_ (blue solid curve), S_0_ (black dotted curve) and S_−1_ (blue thin solid curve). For comparison, the energy minimum in the O4 moiety was set to zero for all S states.

*Case 1. O4 is an H-bond donor (O4=OH^−^) to W539 (“pre-PT” H-bond pattern).* In S_0_ ((Mn1, Mn2, Mn3, Mn4)=(III, IV, III, III)), QM/MM calculations reproduced a short H-bond distance of ∼2.5 Å (2.55 Å, Table 1) for O4–O_W539_ only when O4 was OH^–^ and donated an H-bond to W539 (‘pre-PT’ pattern in Fig. 2; Table 1). In S_1_, the energy profile of the O4–O_W539_ bond (Fig. 3a) resembles an energetically unstable single-well H-bond (Supplementary Fig. 2). These results indicate that matching of the p*K*_a_ values of the donor (that is, O4, Supplementary Fig. 1) and acceptor (that is, W539) moieties occurs in S_1_. Due to the presence of the single-well H-bond (for example, ref. 47), the ‘pre-PT’ H-bond pattern (Fig. 2) was energetically unstable in S_1_ (discussed later). Note that a single-well H-bond has its energy minimum only at the O–O distance of ∼2.5 Å (for example, not 2.6 or 2.7 Å (ref. 43)).

*Case 2. W539 is an H bond donor to O4 (O4=O^2−^) (“post-PT” H-bond pattern).* In the over-reduced state, S_–1_, QM/MM calculations also reproduced the short H-bond distance of ∼2.5 Å for O4–O_W539_ (Table 1) but only when O4 was O^2–^ and when it accepted an H-bond from W539 (‘post-PT’ pattern in Fig. 2).

### PT from OH^–^ at O4 along the O4-water chain

In the QM/MM calculations, OH^–^ at O4 was stable as an H-bond donor to W539 in S_0_ and lower S states (Fig. 3). However, in the S_1_ state, OH^–^ at O4 was energetically unstable due to formation of the single-well H-bond, leading to the release of a proton from OH^–^ at O4 and the formation of O^2–^ at O4. Remarkably, the released proton was stabilized at W1047 in the form of H_3_O^+^, which is 13.5 Å away from O4 (Fig. 1). Overall, the pre-PT pattern (Fig. 2) in the initial state completely transformed into the post-PT pattern (Fig. 2) as a result of PT.

The short O4–O_W539_ in the pre-PT conformation lengthened to ∼2.58 Å in the post-PT pattern in S_0_ or S_1_ (Table 1) from 2.50 Å in the 1.9-Å structure. This longer H-bond in S_0_ or S_1_ is consistent with O4–O_W539_ distances of 2.59 Å in both PSII monomers in the recent radiation-damage-free PSII crystal structure obtained using the X-ray free electron laser (Supplementary Table 1)^36^. These results seem to correspond to a situation in which the free electron laser structure^36^ has the S_0_ or S_1_ state in which the O4 is deprotonated, while in the earlier conventional X-ray diffraction structure (1.9-Å structure)^14^ the Mn_4_CaO_4_ is in a more reduced state and exhibiting a single-well H-bond between O4 and O_W539_.

In the 1.9-Å structure, W1047 has unusually short H-bonds, O_W1047_–O_W545_ (2.50 Å) and O_W1047_–O_W399_ (2.52 Å)^14^. Intriguingly, the short O–O distances were reproduced only when we assumed the post-PT H-bond pattern of the O4-water chain (2.46–2.54 Å, Table 1 and Supplementary Table 2). In general, typical H_2_O…H_3_O^+^ (that is, the Zundel cation) has an unusually short O–O distance of ∼2.4 Å (refs 39, 40). Therefore, the short H-bond distances of W1047 in the 1.9-Å structure imply that W1047 is capable of forming H_3_O^+^. Remarkably, the X-ray free electron laser structure^36^ also has unusually short H-bonds at the corresponding positions (Supplementary Table 1), a further indication that the X-ray free electron laser structure represents the post-PT state. The presence of these single-well H-bonds indicates that local movement of a proton within the O4–O_W539_ bond of ∼2.5 Å results in a sequential downhill PT from O4 to W1047 over a distance of 13.5 Å (Fig. 2 and Supplementary Fig. 3).

### A working model

The results indicate that PT occurs through the O4-water chain and thence towards the lumenal bulk surface via PsbU (Supplementary Discussion). This PT reaction is specifically associated with the S_0_–S_1_ transition. This is supported by the following arguments.

**Figure 4:**
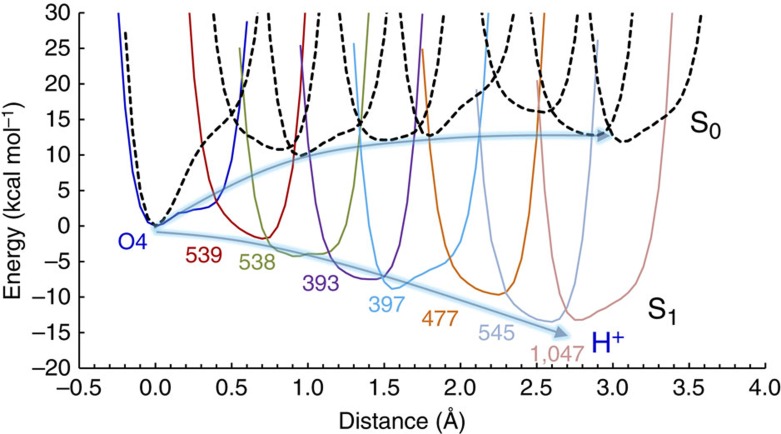
The energy profiles along the PT coordinate for all of the H-bonds along the O4-water chain in the pre-PT conformation; S_1_ (coloured solid curves) and S_0_ (black dotted curves). For comparison, the energy minimum in the O4 moiety was set to zero for all S states. For efficient analysis, the small QM region was adopted. In QM/MM calculations the electrostatic interactions, which are calculated from parametrized partial atomic charge, are sometimes intentionally overestimated. The correct treatment of long-range electrostatics in QM/MM calculations is hard to achieve (see for example, ref. 69) and some improvements are in progress (for example, ref. 70). The present results are to be considered qualitatively.

The O4-water chain is the H-bond network in the 1.9-Å structure^14^ that is directly linked with the O atoms of the Mn_4_CaO_5_ cluster (via the O4–O_W539_ bond). The energy barrier for PT along the O4–O_W539_ bond is clearly the lowest among all the possible Mn_4_CaO_5_ deprotonation sites in the 1.9-Å structure^14^. This should contribute to a smaller activation energy for deprotonation, a property consistent with the known characteristics of the S_0_–S_1_ transition, which includes its rate being insensitive to H_2_O/D_2_O exchange and its activation energy being low compared that of the S_2_–S_3_ transition^21^,^48^,^49^. In the energy profiles of the H-bonds studied here, a proton became energetically more stable as it moved away from Mn_4_CaO_5_, indicating that a downhill PT reaction occurs specifically on the S_0_–S_1_ transition (Fig. 4). The driving force for the PT towards the protein surface in S_1_ is attributed to the increased positive charge on Mn_4_CaO_5_ on the S_0_–S_1_ transition and this conclusion was supported by the observation that lower S-states resulted in the uphill PT reaction (Fig. 4 and Supplementary Fig. 4).

The remarkable linear, eight-water molecular chain acting as Grotthuss-like proton conduit, which is reported here, might be expected to work with very fast kinetics. However, proton release on this step occurs relatively slowly (tens of microseconds^21^). This is easily understandable since the electron transfer step occurs before H^+^ release and is thus rate-limiting^21^.

### Energetics of proton release from O5

**Figure 5:**
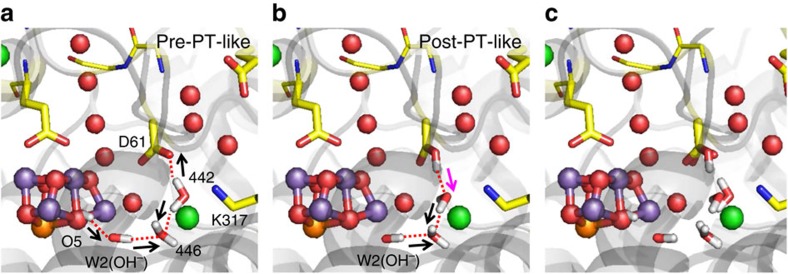
QM/MM optimized geometries. (**a**) pre-PT-like, (**b**) post-PT-like and (**c**) both conformations. Initial donor-to-acceptor orientations are indicated by black arrows, whereas altered donor-to-acceptor orientations in the post-PT conformation are indicated by magenta arrows.

**Figure 6:**
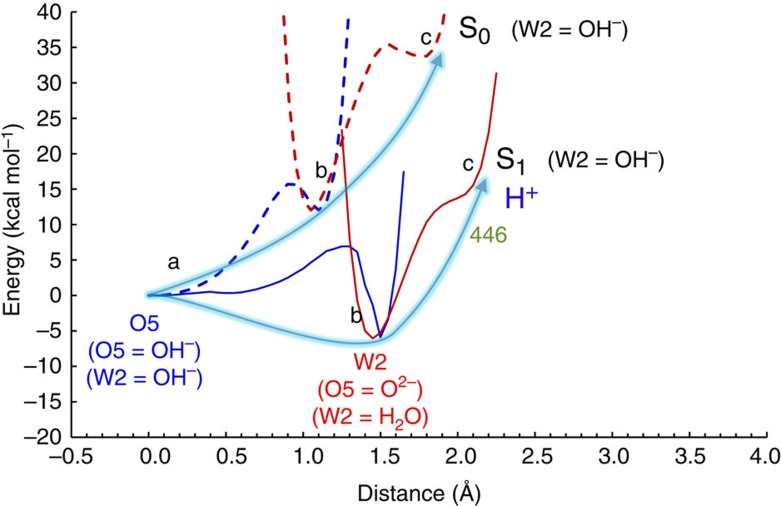
The energy profiles along the PT coordinate for all of the H-bonds along the O5 path. In the pre-PT conformation; S_1_ (solid curves) and S_0_ (dotted curves) for W2=OH^−^ (see Supplementary Fig. 6 for W2=H_2_O). For comparison, the energy minimum in the O5 moiety was set to zero for all S states. Labels **a**–**c** correspond to the states in Supplementary Fig. 5a–c, respectively.

**Figure 7:**
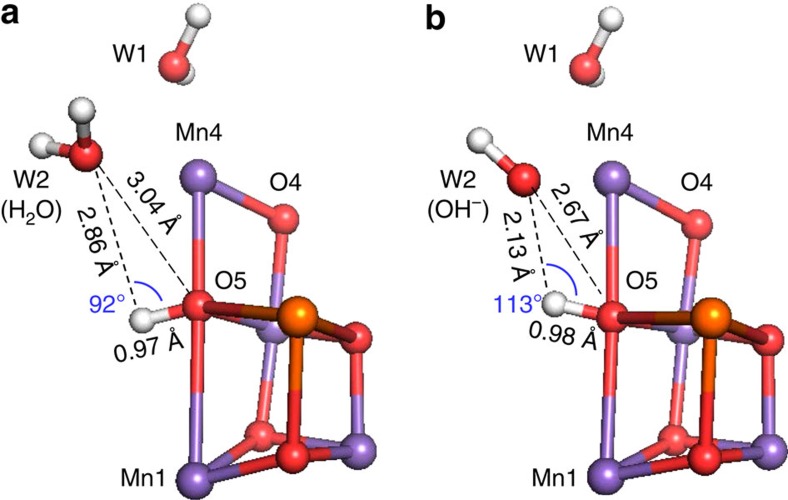
H-bond patterns of the O5 and W2 moiety in the QM/MM geometry. (**a**) W2=H_2_O and (**b**) W2=OH^−^. For clarity, only the Mn_4_CaO_5_ cluster, W1, and W2 are shown.

In the present work we also analysed the energy profiles of the PT along the O5 path that proceeds from O5 to D1-Asp61 via the water molecules, W2, W446 and W442 (Fig. 5). W2 is the only possible proton acceptor from OH^–^ at O5 when the original geometry of the 1.9-Å (ref. 14) or X-ray free electron laser^36^ structure is maintained (see below and Supplementary Discussion). When OH^–^ at W2 becomes H_2_O by accepting the proton from OH^–^ at O5 (Fig. 6b and Supplementary Fig. 5b), W2 cannot release a proton easily to its H-bond acceptor W446 (Fig. 6b,c and Supplementary Fig. 5b,c, see Supplementary Discussion for details), thus causing a significant energy barrier for further PT (Fig. 6). Note that the PT from O5 to W2 is more unlikely when W2=H_2_O (Supplementary Fig. 6) due to the even less appropriate H-bond angle for O5…W2 (Fig. 7).

For a PT, formation of a proton conducting wire (that is, proton donor and acceptor pairs), which is itself an activated process, must occur first^50^. The activation-less ‘pre-organized’ proton conducting wire possesses well-arranged water groups along the O4-water chain (Fig. 2e) and appears to be achieved by the ‘pre-organized protein dipole^51^’ of PSII. A corresponding feature is absent (for example, a partial H-bond network) in the O5 path (Fig. 5), suggesting that a sequential downhill PT from O5 is unlikely. It seems clear that deprotonation of a putative OH^–^ at O5 is less likely than OH^–^ at O4. The activation barrier of the O5 path appears to remain high in S_1_ (Fig. 6); it might be possible however that this path becomes active in proton conduction in higher S states.

### Alternative O5 deprotonation models

Although some studies have suggested or assumed that O5 (=OH^–^) deprotonation occurs on the S_0_–S_1_ transition^33^,^34^,^35^, it should also be noted that O5 has no direct-H-bond partner in the original geometry of the 1.9-Å structure^14^ (Fig. 7) and in the free electron laser structure^36^. Recent QM/MM studies by Pal *et al*.^34^ also demonstrated that OH^–^ at O5 has no direct-H-bond partner in their S_0_ model; this implies that the ‘energy barrier’ for PT with O5 must be high. Indeed, Pal *et al*. demonstrated that O5 deprotonation only occurred when using ‘a small model of the oxidized S_0_′ state’ (that is, not ‘the QM/MM S_0_ model’, see supplementary Fig. 7b in ref. 34), in line with our conclusions.

Siegbahn put forward a model in which O5 (=OH^–^) deprotonation occurred on the S_0_–S_1_ transition by assuming an additional water molecule near O5 in density functional theory (DFT) calculations^35^, a water molecule that is not visible not only in the original 1.9-Å structure^14^ but also in the recent radiation-damage-free PSII crystal structure^36^. This extra water molecule was positioned so that it could accept an H-bond from O5 (ref. 35). An appropriately oriented proton acceptor for O5 would significantly decrease the energy barrier for release of a proton from O5. In this case however, the actual 1.9-Å structure shows that the water molecule cannot be located at the corresponding position due to the presence of a conserved hydrophobic amino acid side-chain, D1-Val185 (Cγ_Val185_−O_water_=∼2.1 Å in Supplementary Fig. 7). This feature is also present in the free electron laser structure, which is assumed to be in the S_1_ state^36^. In the O5 deprotonation model, D1-Val185 was not included in the calculation and hence was absent from the model^35^. Furthermore, the O5 deprotonation model also included a significant structural modification in the vicinity of the H-bond accepting water: the side-chain of D1-His332 was twisted (by ∼90^°^) along the Mn1-Nε_His332_ axis^35^ compared with its position in the 1.9-Å structure. These two structural differences compared with the reference crystal structure thus allowed the water to be located close to O5 without the steric repulsion that would have occurred in the unmodified 1.9-Å structure (Supplementary Fig. 7). It could be argued *ad hoc* that a conformational change may occur resulting in the situation modelled. However the most recent unreduced structure of the enzyme shows no sign of such changes^36^, so arguments for such a conformational change are not compelling. Without specific justification for the ∼90° twist of the Mn-ligated D1-His332 side-chain and the relocation of the conserved hydrophobic D1-Val185 side-chain away from the O5 moiety, it seems unjustified to place a water molecule at the position required for it to accept an H-bond from O5 for modelling the S_0_ and S_1_ transition.

The O5 deprotonation model also showed significantly large deviations of the atomic coordinates from those of the original 1.9-Å structure^35^ not only for D1-His332 (root-mean-square deviation=1.28 Å) but also for CP43-Arg357 (1.19 Å) and D1-Glu189 (0.51 Å, Supplementary Table 3). Furthermore, the H-bond partner of O4, that is, W539, and the entire O4-water chain were also absent in the earlier model^35^. These changes and omissions are expected to affect the resulting energy of the system in that model.

Overall then based on the current data, it seems clear that the deprotonation of a OH^–^ at O5, as suggested earlier^34^,^35^, is less likely than deprotonation of OH^–^ at O4, as suggested in the present work.

### Deprotonation of the substrate oxygen and protonation of O4

It has been proposed that the exchangeable μ-oxo bridge is likely to be O5 (refs 24, 29, 33, 52; see also relevant articles^19^,^20^ published before the detailed 1.9-Å structure^14^), and thus O5 is a plausible candidate for the slow exchanging substrate water molecule W_s_ (ref. 53). This is not incompatible with the presence of protonated O4 in S_0_: it is possible that the liberation of O_2_ on formation of S_0_ is associated with the release of a proton from the substrate ending up on μ-oxo bridge O4. A similar protonation of a μ-oxo bridge on liberation of O_2_ has been proposed in manganese catalases^54^.

### The O4-water chain and the low S_0_/S_1_ redox potential

The conversion of the S_0_ state into the S_1_ state^55^ due to its oxidation by TyrD^·^ showed that the S_0_/S_1_ redox potential is lower than that of TyrD redox potential^56^, the latter was estimated to be ∼720–760 mV (refs 57, 58). Given that all the S_0_ present is converted to S_1_ by TyrD^·^, the S_0_/S_1_ redox potential can be taken to be ≤700 mV assuming the estimates for TyrD redox couple are reasonable. In contrast, the S_1_/S_2_ and S_2_/S_3_ redox potentials are clearly higher since they oxidize TyrD^56^ and they have been suggested to be ∼900–950 mV (ref. 58). Thus, the barrier-less deprotonation and the subsequent downhill PT reaction occurring on the S_0_–S_1_ transition may contribute to the uniquely low redox potential of this redox couple, compared with those of the other S-state transitions. As demonstrated by Warshel and coworkers^47^,^51^, low-barrier H-bonds (including single-well H-bonds), where the p*K*_a_ difference for the donor and acceptor moieties is nearly zero (that is, less polarized), are particularly unstable in polar protein environments. An O4–O_W539_ H-bond will become a very unstable low-barrier H-bond upon oxidation to S_1_, resulting in the release of the proton via the O4-water chain (Table 1). This is consistent with the observation that S_1_ does not spontaneously back-react to form S_0_ (ref. 59). PT may proceed through different pathways depending on the S-state transitions^5^,^6^,^7^. Intriguingly, it has been reported that the rate constant for the S_0_–S_1_ transition is essentially pH-independent, whereas that for S_2_–S_3_ transition is pH-dependent^21^,^48^,^49^. Ionizable groups are more likely to be involved in a PT pathway for a pH-dependent process than a pH-independent process; this fits with the suggestion that the uncharged O4-water chain may be active in the S_0_–S_1_ transition.

### Changes in Mn–Mn distances by Mn oxidation/deprotonation

**Figure 8:**
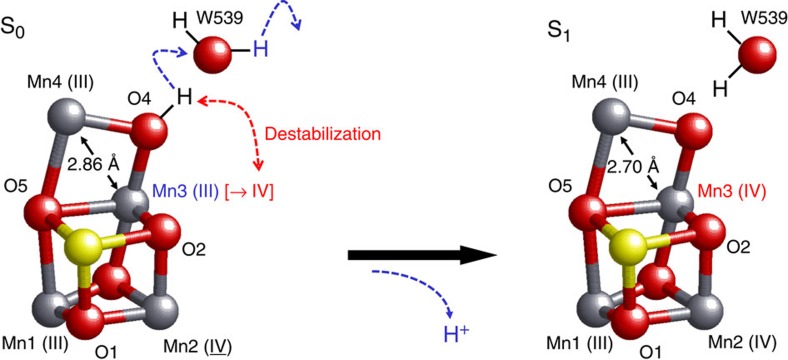
Oxidation states of the four Mn ions and protonation states of the O atoms. The release of a proton from O4 occurs due to oxidation at O4 via the single-well H-bond with W539 upon formation of S_1_.

On proton release from O4, the Mn ion undergoing oxidation was Mn3 (Fig. 8). We note that the Mn undergoing oxidation is unlikely to be Mn4 because it appears to be the most reduced site, that is, it has the highest potential (for example, refs 29 and 60). Our results suggest that on formation of S_1_, oxidation of Mn3 from the III valence to IV favours the release of the proton from OH^−^ at O4 (Fig. 8). From ENDOR, electron paramagnetic resonance (EPR) and simulation of the EXAFS, it has been proposed that a Mn–Mn distance of ∼2.85 Å in the S_0_ state is decreased to ∼2.7 Å in the S_1_ state (2.83–2.73 Å (ref. 16), 2.85–2.72 Å (ref. 19) or 2.85–2.75 Å (ref. 20)). In the present calculations, Mn3–Mn4 was the only distance that was decreased by ∼0.1 Å from 2.86 Å in S_0_ to 2.70 Å in S_1_ (Table 2 and Supplementary Table 4). Note that the corresponding change was 2.90, a slightly longer distance, to 2.71 Å when O5 was assumed to be the deprotonation site^34^. These results suggest that the decreased Mn–Mn distance reported in EXAFS studies^16^,^17^,^18^,^19^,^20^,^21^ corresponds to the Mn3–Mn4 distance, which is decreased by deprotonation of O4 in the S_0_–S_1_ transition.

Mn3–Mn4 is a key distance that can allow us to evaluate the reduction state of PSII crystal structures with respect to geometries obtained from ENDOR, EPR or simulation of the EXAFS^16^,^17^,^18^,^19^,^20^,^21^ in both S_0_ and S_1_. The Mn3–Mn4 distance of 2.87 Å in the free electron laser structure^36^, which was reproduced (2.91 Å with OH^–^ at O5 in S_1_) in QM/MM studies by Shoji *et al*.^61^ is close to the distance of S_0_ (∼2.85 Å), not the distance of S_1_ (∼2.73 Å), obtained from ENDOR, EPR and EXAFS. This is in line with the presence of significant amount of the reduced S_0_ state suggested from recent QM/MM studies by Askerka *et al*.^38^ It seems likely that the geometry of the free electron laser structure does not represent a pure S_1_ state detected ENDOR, EPR and EXAFS^16^,^17^,^18^,^19^,^20^,^21^ and is probably in a more reduced state.

An argument for the presence of a hydroxide ion at O5 for the free electron laser structure proposed by Suga *et al*.^36^ was the observation of significantly lengthened distances between O5 and the adjacent Mn ions. The presence of a hydroxide ion at O5 was considered likely because recent QM/MM studies by Shoji *et al*.^61^ have reproduced two of the three significantly lengthened distances between O5 and the adjacent Mn ions, Mn1–O5 (2.70 Å (ref. 36) and 2.73 Å (ref. 61)) and Mn4–O5 (2.33 Å (ref. 36) and 2.34 Å (ref. 61)), by assuming OH^–^ at O5 in S_1_ (ref. 61). However, it should also be noted that one of the three significantly lengthened distances in the free electron laser structure, Mn3–O5, could not be reproduced in this study^61^; in fact Mn3–O5 was significantly shortened in the QM/MM geometry (2.20 Å (ref. 36) to 1.96 Å (ref. 61)). Indeed, the 1.9 Å structure has an even longer Mn3–O5 distance of 2.38 Å (ref. 14). Notably, a similar Mn3–O5 distance of 2.37 Å was obtained, assuming reduced Mn(II) for Mn4 in DFT studies by Petrie *et al*.^62^.

These features of the free electron laser structure, (i) the lengthened Mn3–Mn4 distance with respect to S_0_ of ENDOR, EPR and EXAFS^16^,^17^,^18^,^19^,^20^,^21^ and (ii) the lengthened Mn3–O5 distance with respect to the QM/MM geometry (S_1_ with OH^–^ at O5 (ref. 61)), suggest that Mn_4_CaO_5_ of the free electron laser structure is probably reduced and cannot be simply explained as representing a single oxidation (and protonation) state.

## Discussion

Based on the findings reported here, we are able to propose a mechanism for PT along the O4-water chain. (Note that the role of the O4-water chain as a proton channel as proposed here does not exclude a role as a substrate channel on other steps of the cycle.) On the S_0_–S_1_ transition deprotonation of a μ-hydroxo group at the O4 position occurs due to oxidation of Mn3(III) to Mn3(IV) (Fig. 8). This deprotonation results in a decrease in the Mn3–Mn4 distance from 2.86 to 2.70 Å (Table 2 and Supplementary Table 4); a similar decrease in the Mn–Mn separation has been reported in ENDOR, EPR or EXAFS studies^16^,^19^,^20^. The nature of the O4-water chain, being composed exclusively of water molecules, is consistent with and may explain the pH-independence of PT in the S_0_–S_1_ transition 21,48,49. At the start of the O4-water chain, the proton in the barrier-less O4–O_W539_ H-bond is likely to move away from the positively charged S_1_ when it forms. Thus, the O4–O_W539_ H-bond is energetically less stable in S_1_, resulting in the sequential (Fig. 2) and downhill (Fig. 4) PT reaction. The presence of the O4-water chain may explain why formation of the S_1_ state is less inhibited by Cl^–^ depletion^63^. It may also explain the apparently irreversibility of S_0_–S_1_ step^59^ and the redox potential of S_0_/S_1_ being uniquely low, lower than the TyrD redox potential^56^,^58^, resulting in the conversion of S_0_–S_1_ in the dark^55^,^56^.

It has been suggested that in bacteriorhodopsin, the PT occurs to the Schiff base along a chain of three water molecules, by transforming from the pre-PT to post-PT patterns^64^. As far as we are aware the O4-water chain is the longest PT channel of water molecules (∼8-water molecules) identified in protein crystal structures. This unusually long and straight water chain, which seems to function as a PT pathway in the water-oxidizing enzyme, is worthy of study in its own right.

## Methods

### Coordinates and atomic partial charges

The atomic coordinates of PSII were taken from the X-ray structure of PSII monomer unit ‘A’ of the PSII complexes from *Thermosynechococcus vulcanus* at a resolution of 1.9 Å (Protein Data Bank (PDB) code, 3ARC)^14^. Hydrogen atoms were generated and energetically optimized with CHARMM^65^, whereas the positions of all non-hydrogen atoms were fixed, and all titratable groups were kept in their standard protonation states (that is, acidic groups were ionized and basic groups were protonated). For the QM/MM calculations, we added additional counter ions to neutralize the entire system. Atomic partial charges of the amino acids were adopted from the all-atom CHARMM22 (ref. 66) parameter set. The atomic charges of cofactors were taken from our previous studies of PSII^43^.

### QM/MM calculations

We employed the electrostatic embedding QM/MM scheme, in which electrostatic and steric effects created by a protein environment were explicitly considered, and we used the Qsite^67^ programme code, as used in previous studies^43^. We employed the unrestricted DFT method with the B3LYP functional and LACVP** basis sets. To analyse H-bond potential-energy profiles of the O4–O_W539_ bond, the QM region was defined as the Mn_4_CaO_5_ cluster (including the ligands), water molecules shown in Fig. 1, side-chains of D1-Asp61, D1-Asn338, D2-Asn350, and CP43-Thr335, and backbones of D1-Asp61, D1-Asn335, D1-Ala336, D2-Asn350 and CP43-Gly338, whereas other protein units and all cofactors were approximated by the MM force field. To analyse all of the H-bonds along the O4-water chain (Fig. 1) in the pre-PT conformation, a slightly smaller QM region was defined as (the Mn_4_CaO_5_ cluster (including the ligands), water molecules shown in Fig. 1 and the side-chain of CP43-Thr335) for efficiency (small QM). The two QM regions did not essentially alter the results and conclusions. The resulting geometries were essentially identical regardless of the size of the QM region (Supplementary Tables 2 and 4). The results obtained on the basis of the large QM region were described in the main text. To analyse all of the H-bonds along the O5 path (Fig. 1) in the pre-PT-like conformation, the QM region was defined as (the Mn_4_CaO_5_ cluster (including the ligands), water molecules of W1, W2, W3, W4, W539, W538, W446, and W442, side-chains of D1-Asp61, D1-Asn181, and D2-Lys317 and a chloride ion (Cl^-^1)). To analyze H-bond patterns of the O5 and W2 moiety (Fig. 7), we used the QM region same as that in ref. 68. The geometries were refined by constrained QM/MM optimization. Specifically, the coordinates of the heavy atoms in the surrounding MM region were fixed at their original X-ray coordinates, while those of the H atoms in the MM region were optimized using the OPLS2005 force field. All of the atomic coordinates in the QM region were fully relaxed (that is, not fixed) in the QM/MM calculation. Note that the resulting atomic coordinates of the QM region essentially did not alter upon full relaxation of the entire atoms (including heavy atoms) in the surrounding MM region^68^. The Mn_4_CaO_5_ cluster was considered to be ferromagnetically coupled; for example, the total spin *S*=7 in S_1_ and the resulting Mn oxidation state (Mn1, Mn2, Mn3, Mn4)=(III, IV, IV, III) (see Supplementary Discussion for further details). The potential-energy profiles of H-bonds were obtained as follows: first, we prepared the QM/MM optimized geometry without constraints and used the resulting geometry as the initial geometry. The H atom under investigation was then moved from the H-bond donor atom (O_donor_) towards the acceptor atom (O_acceptor_) by 0.05 Å, after which the geometry was optimized by constraining the O_donor_–H and H–O_acceptor_ distances. The energy of the resulting geometry was calculated. This procedure was repeated until the H atom reached the O_acceptor_ atom. See Supplementary Data 1 for the atomic coordinates of the QM region (that is, Mn_4_CaO_5_). As discussed later, OH^–^ at O5 has no direct-H-bond in the protein environment of PSII. Thus, to analyse the PT from O5, we assumed W2 (O5…O_W2_=3.1 Å (ref. 14)) as the plausible proton acceptor, and analysed the energy profile by constraining the H–O_W2_ distance. We also calculated the ^1^H NMR chemical shift for OH^−^ at O5 (see Supplementary Methods and Supplementary Discussion).

## Additional information

**How to cite this article:** Saito, K. *et al.*, Energetics of proton release on the first oxidation step in the water-oxidizing enzyme. *Nat. Commun.* 6:8488 doi: 10.1038/ncomms9488 (2015).

## Supplementary Material

Supplementary Information Supplementary Figures 1-8, Supplementary Tables 1-4, Supplementary Discussion, Supplementary Methods and Supplementary References

Supplementary Data 1 Atomic coordinates for QM/MM calculations

## Figures and Tables

**Table 1:** H-bond geometries (in Ångstroms) of the O4 site.

			**pre-PT (O4=OH** ^−^ **)**			**post-PT (O4=O** ^2−^ **)**	
**High (** ***low*** **)** *		**S**_**1**_ **(*****S***_***3***_**)**	**S**_**0**_ **(*****S***_***2***_**)**	**S**_**−1**_ **(*****S***_***1***_**)**	**S**_**1**_ **(*****S***_***3***_**)**	**S**_**0**_ **(*****S***_***2***_**)**	**S**_**−1**_ **(*****S***_***1***_**)**
Mn1–4	**X-ray**	III, IV, IV, III	III, IV, III, III	III, III, IV, II	III, IV, IV, III	III, IV, III, III	III, III, IV, II
O4–539	2.50	**2.45**	**2.55**	2.64	2.58	2.57	**2.49**
539–538	2.77	**2.55**	2.59	2.61	2.78	2.72	2.76
538–393	2.71	2.60	2.64	2.66	2.70	2.68	2.65
393–397	2.73	2.62	2.64	2.67	2.75	2.72	2.71
397–477	2.69	2.83	2.83	2.87	2.68	2.66	2.64
477–545†	2.87	2.72	2.71	2.71	2.71	2.70	2.69
545–1047	2.50	2.61	2.61	2.60	2.66	2.66	2.66
1047–399	2.52	2.71	2.70	2.68	**2.53**	**2.54**	2.56
RMSD: Mn_4_Ca		0.11	0.20	0.18	0.16	0.24	0.16
RMSD: Mn_4_CaO_5_		0.27	0.31	0.30	0.30	0.30	0.30

RMSD, root-mean-square deviation.

^*^High oxidation state model in normal script (low oxidation state model in italics and parenthesis^15^).

^†^The large deviation of the W477–W545 length from the 1.9-Å structure^14^ originates from the poorer density specifically for W477 in the crystal (Supplementary Fig. 8). In the O4-water chain, W477 is the only water molecule that does not donate an H-bond to a backbone C=O but accepts an H-bond from the backbone NH of CP43-G353 (Fig. 1) and this seems to be associated with the greater disorder.

O5=O^2−^; O4=OH^–^ in pre-PT and O^2−^ in post-PT. RMSD of the optimized heavy atoms with respect to those of the 1.9-Å structure. Short H-bond distances (< ∼2.5 Å) are in bold.

**Table 2:** Mn–Mn distances (Å).

	**S**_**0**_ **: pre-PT (O4=OH** ^−^ **)**	**S**_**1**_ **: post-PT (O4=O** ^2−^ **)**
Mn1–4	III, IV, IV, III	III, IV, IV, III
Mn1–Mn2	2.75	2.75
Mn2–Mn3	2.76	2.76
Mn1–Mn3	3.36	3.28
Mn3–Mn4	2.86	2.70*
Mn1–Mn4	4.74	4.81

^*^Essentially consistent with the value of 2.71 Å in S_1_ (O4 and O5=O^2−^) in other studies (for example, ref. 34).
