# Supplementary material for: Energetics of proton release on the first oxidation step in the water-oxidizing enzyme
Source: Nat Commun. 2015 Oct 7;6:8488. doi: 10.1038/ncomms9488 (PMC4617610; doi:10.1038/ncomms9488)
Supplement: Supplementary Information — Supplementary Figures 1-8, Supplementary Tables 1-4, Supplementary Discussion, Supplementary Methods and Supplementary References [file ncomms9488-s1.pdf]

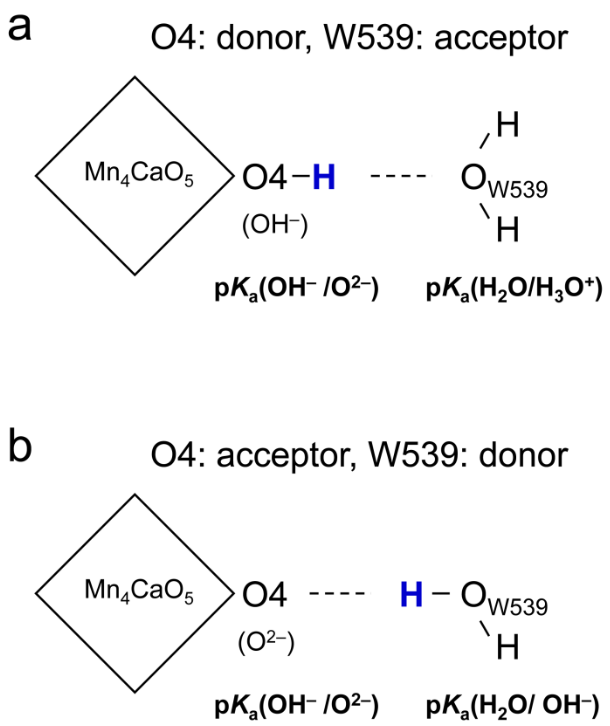

Supplementary Figure 1. Relevant pK_a species of the H-bond donor/acceptor moieties in the O4–O_{W539} bond. (a) pre-PT; (b) post-PT.

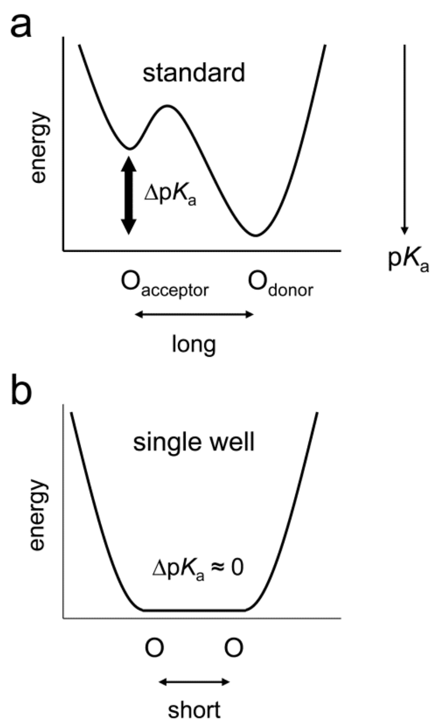

Supplementary Figure 2. An overview of typical potential-energy profiles. (a) standard H-bonds (asymmetric double-well); (b) single-well H-bonds.

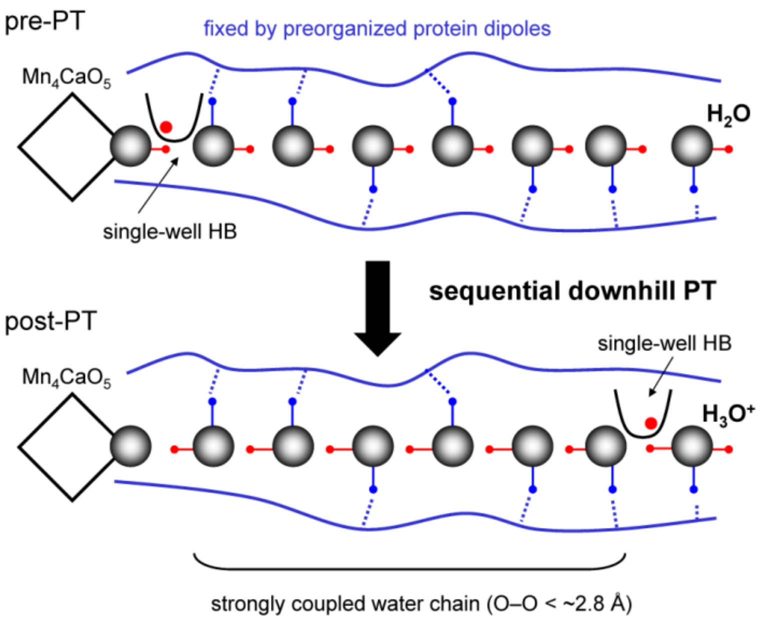

Supplementary Figure 3. Sequential downhill proton transfer (PT) process along the water chain. Altered and unaltered H-bonds are indicated by red and blue lines and balls, respectively. The black curves at the entrance of the chain in the pre-PT H-bond pattern and at the terminus in the post-PT H-bond pattern represent single-well H-bonds.

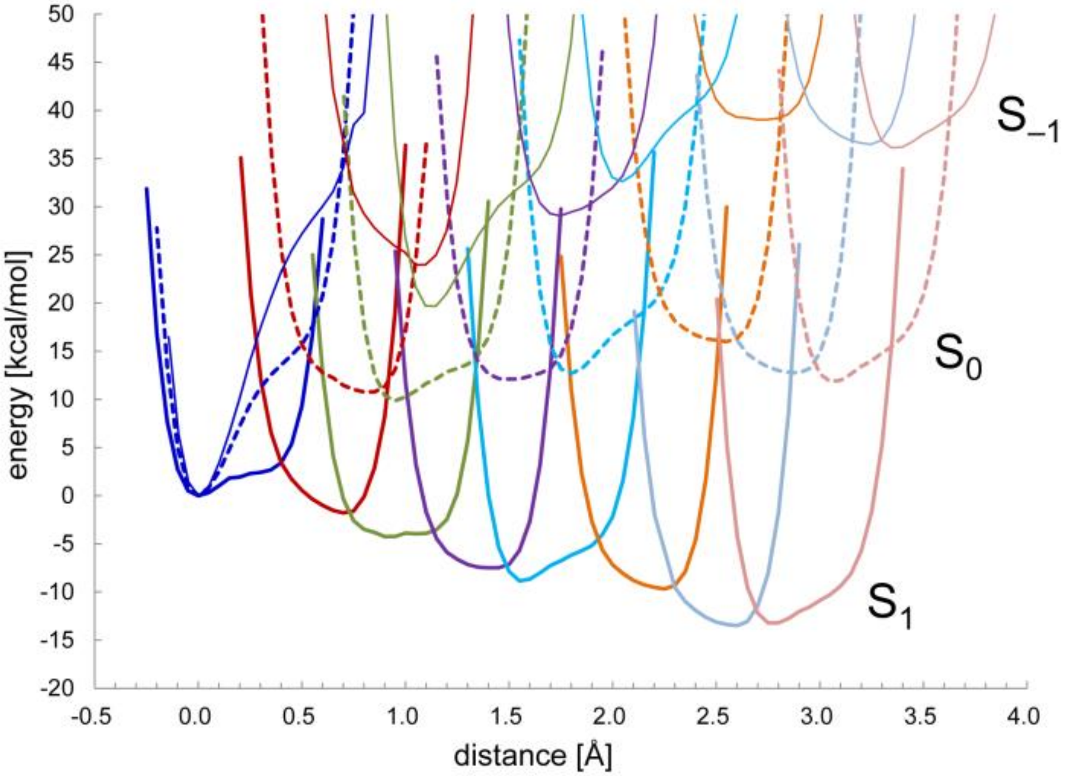

Supplementary Figure 4. The energy profiles along the proton transfer coordinate for all of the H-bonds along the O4-water chain in the pre-PT conformation; S_1 (solid curves), S_0 (dotted curves), and S_{-1} (thin solid curves). For comparison, the energy minimum in the O4 moiety was set to zero for all S states.

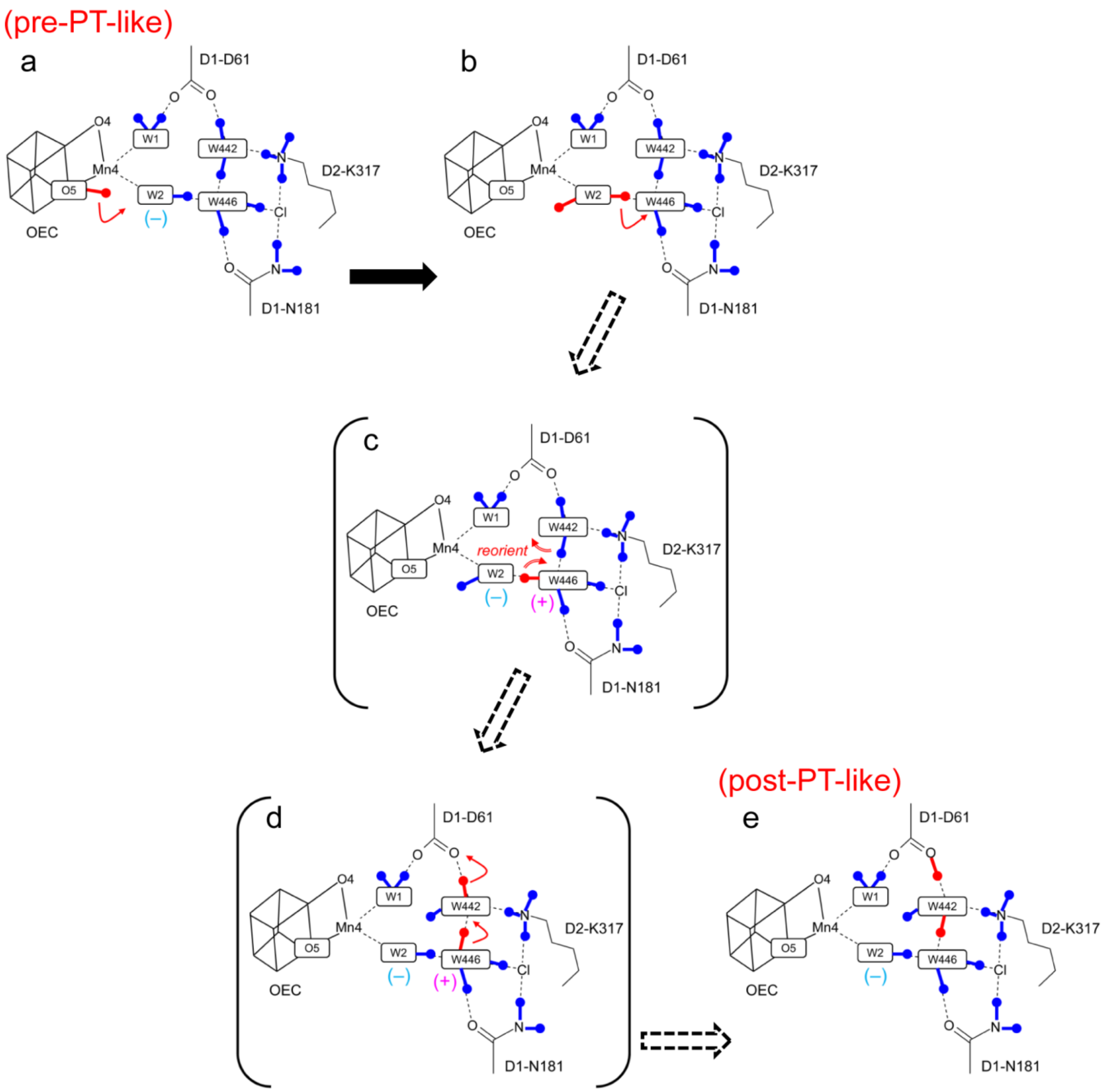

Supplementary Figure 5. H-bond pattern change of the O5 path required for a release of a proton from O5 to D1-Asp61 via water molecules near Cl^-. In contrast to the O4-water chain, a sequential PT from O5 via a water molecule near Cl^- (i.e., W446) is geometrically not allowed: the pre-PT-like and post-PT-like patterns correspond to the pre-PT and post-PT patterns in the O4-water chain, respectively. Altered and unaltered H-bonds are indicated by red and blue lines and balls, respectively.

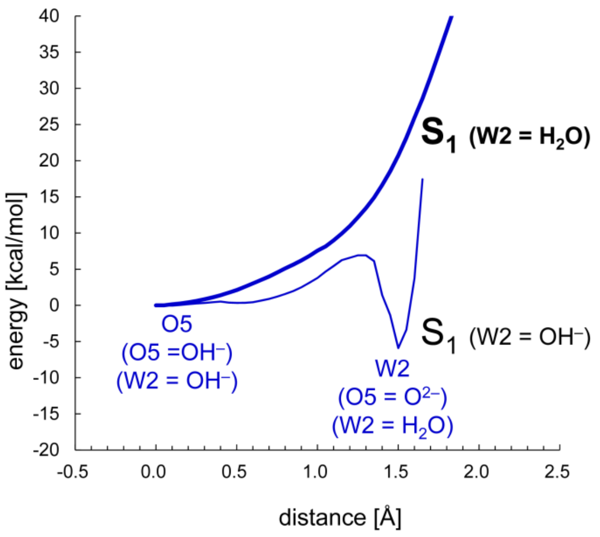

Supplementary Figure 6. The energy profiles along the proton transfer coordinate for all of the H-bonds along the O5 path in the pre-PT conformation in S1; when W2 = H_2O (thick solid curve) or W2 = OH^- (solid thin curve). For comparison, the energy minimum in the O5 moiety was set to zero for all S states.

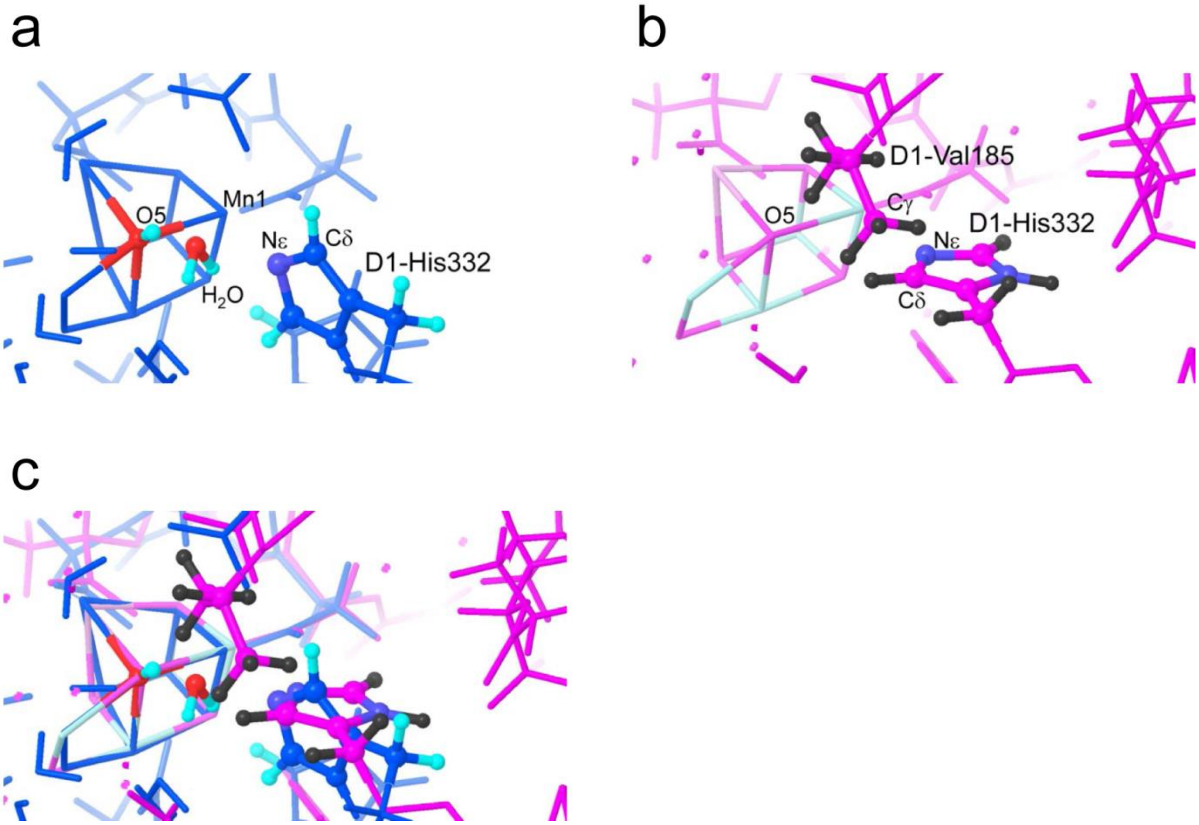

Supplementary Figure 7. Location of the additional water molecule near O5 and orientation of the D1-His332 side chain. (a) Siegbahn's model [30]. (b) The corresponding position of the additional water molecule is occupied by the hydrophobic side chain of conserved D1-Val185 in the PSII crystal structure [9]. (c) Superpositioned geometries with respect to the Mn_4CaO_5 coordinates: the additional water molecule would suffer from steric repulsion with the original orientations of the D1-Val185 and D1-His332 side chains.

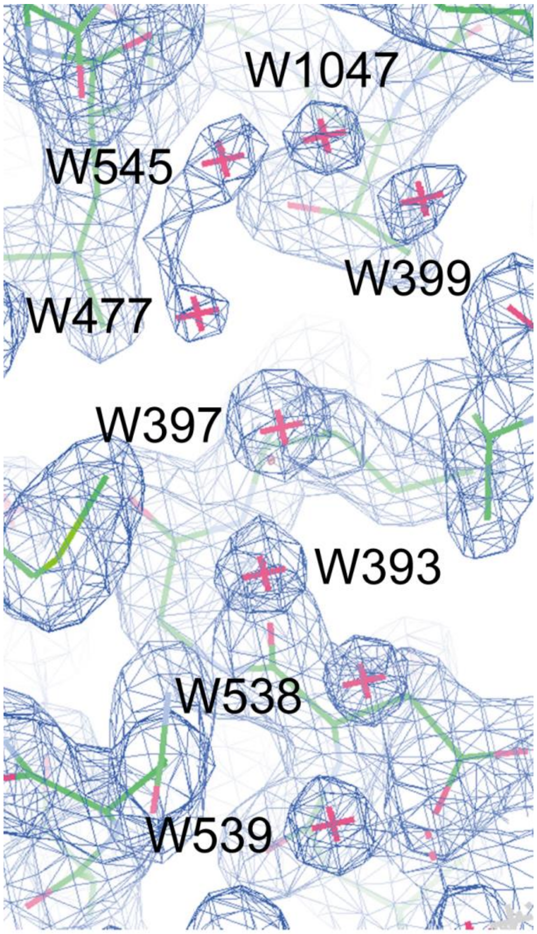

Supplementary Figure 8. The electron density map in the O4-water chain region.

Supplementary Table 1. Distances (in Ångstroms) of the oxygen atoms along the O4-water chain in the PSII crystal structures. n.d.; not determined.

**3ARC (A)**		**3ARC (B)**		**4UB6 (A)**		**4UB6 (B)**		**4UB8 (A)**		**4UB8 (B)**	
**water**	**dist.**	**water**	**dist.**	**water**	**dist.**	**water**	**dist.**	**water**	**dist.**	**water**	**dist.**
O4		O4		O4		O4		O4		O4	
	2.50		2.43		2.59		2.59		2.61		2.69
w539A		w539a		w567A		w525a		w568A		w524a	
	2.77		2.81		2.78		2.92		2.69		2.80
w538C		w538c		w665C		w1060c		w660C		w661c	
	2.71		2.69		2.59		2.70		2.57		2.96
w393A		w1253a		w542A		w619a		w543A		w620a	
	2.73		2.80		2.78		2.64		2.80		2.56
w397A		w1255a		w546A		w621a		w547A		w622a	
	2.69		2.74		2.69		3.22		2.84		3.04
w477C		w477c		w612C		w1010c		w608C		w608c	
					2.78				2.80		2.78
(n.d.)	2.87	(n.d.)	2.80	w806C		(n.d.)	2.80	w796C		w787c	
					2.93				2.67		2.95
w545A		w545c		w696C		w635a		w578C		w634A	
	2.50		2.59		2.25		2.66		2.19		2.43
w1047C		1047c		w757C		w1154c		w752C		w752c	
	2.52		2.59		2.65		2.62		2.66		2.81
w399A		1257a		w548A		w623a		w549A		w624a	

sites : 8	8	9	8	9	9

Supplementary Table 2. H-bond geometries (in Ångstroms) of the O4 site in Mn_4CaO_5 in different protonation states.

O5 = O^{2-}; O4 =OH^- in pre-PT and O^{2-} in post-PT.

QM region = small, [the Mn_4CaO_5 cluster (including the ligands), water molecules shown in Figure 1, and the side chain of CP43-Thr335].

O4	X-ray		(pre-PT) OH^-			(post-PT) O^{2-}		
		High *(Low)*[a]	S_1 (S_3)	S_0 (S_2)	S_{-1} (S_1)	S_1 (S_3)	S_0 (S_2)	S_{-1} (S_1)
Mn1			III	III	III	III	III	III
Mn2			IV	IV	IV	IV	IV	III
Mn3			IV	III	III	IV	III	IV
Mn4			III	III	II	III	III	II
O4-539	2.50		**2.52**	2.59	2.64	2.63	2.57	**2.49**
539-538	2.77		2.55	2.62	2.62	2.74	2.75	2.73
538-393	2.71		2.63	2.66	2.70	2.69	2.66	2.64
393-397	2.73		2.62	2.64	2.68	2.72	2.70	2.68
397-477	2.69		2.72	2.73	2.75	2.61	2.60	2.59
477-545	2.87		2.62	2.62	2.61	2.59	2.59	2.59
545-1047	2.50		2.61	2.61	2.60	**2.46**	**2.46**	**2.47**
1047-399	2.52		2.71	2.70	2.70	**2.50**	**2.49**	**2.48**
RMSD								
Mn_4Ca			0.09	0.19	0.15	0.13	0.24	0.24
Mn_4CaO_5			0.27	0.29	0.28	0.28	0.31	0.32

[a] High oxidation state model in normal script (*low oxidation state model in italics and parenthesis* [12]).

Supplementary Table 3. Root-mean-square deviation of the optimized heavy atoms with respect to those of the 1.9-Å structure [9] (Å). Values in bold indicate significantly large deviation of Siegbahn's model [30].

	S_0^{-1}; Siegbahn [a]	S_0, pre-PT; Saito et al. [b]
D1-Asp170	0.28	0.29
**D1-Glu189**	**0.51**	**0.14**
**D1-His332**	**1.28**	**0.20**
D1-Glu333	0.21	0.25
D1-Asp342	0.26	0.18
D1-Ala344	0.35	0.23
CP43-Glu354	0.17	0.18
**CP43-Arg357**	**1.19**	**0.10**
water	0.38	0.35
Mn_4Ca	0.19	0.20
Mn_4CaO_5	0.22	0.31
**entire QM**	**0.66**	**0.24**

[a] See Ref. [30].

[b] This work (large QM).

Supplementary Table 4. Mn–Mn distances (Å): small QM.

Protonation of (O4, O5)	(OH−, O2−)<br>S0: pre-PT	(O2−, O2−)<br>S1: post-PT
Mn1, Mn2, Mn3, Mn4	III, IV, IV, III	III, IV, IV, III
Mn1-Mn2	2.75	2.76
Mn2-Mn3	2.79	2.76
Mn1-Mn3	3.36	3.23
Mn3-Mn4	2.86	2.72[a]
Mn1-Mn4	4.73	4.82

[a] Essentially consistent with the value of 2.71 Å in S_1 (O4 and O5 = O^{2-}) in studies by Pal et al [8].

Supplementary Discussion

Absence of an H-bond of a putative OH^- at O5 in the PSII protein environment.

It was suggested that a strong H-bond results in a more downfield ^{1}H NMR chemical shift (δ_H). According to the classification of H bonds by Jeffrey [1] or Frey [2], "single-well H bonds" (or "symmetrical H bonds" [3]) are very short typically with O–O distances of 2.4 to 2.5 Å and display ^{1}H NMR chemical shifts of 20 to 22 ppm [2]. "LBHBs" (or "asymmetric H bonds" [3]) are longer, 2.5 to 2.6 Å with δ_H of 17 to 19 ppm [2]. "Weak H-bonds" are longer still, with δ_H of 10 to 12 ppm [2]. It has been demonstrated that δ_H values can be calculated with good precision, with calculated values matching the experimentally measured values with discrepancies of ~1 ppm or less for organic compounds [4] or proteins [4,5] in particular for H-bonds with δ_H > ~12 ppm [6]. In particular for short H-bonds where the proton essentially lies in-line with the donor and acceptor atom (e.g., O–O < ~2.6 Å), (i) O…H…O-bond geometries and the NMR chemical shifts can also be evaluated by the correlation proposed by Limbach et al. [7], (ii) QM/MM calculations can nicely reproduce measured δ_H values (e.g., [4,5]). Thus, the characteristics of H-bonds can also be evaluated by δ_H. As far as we are aware, detailed chemical shifts for key H-bonds are not reported for PSII. But, it is possible to calculate δ_H for each H-bond in PSII on the basis of the crystal structure and to evaluate its characteristics.

Pal et al. considered W2 as a candidate as the H-bond acceptor to a putative O5 hydroxyl group [8]. The QM/MM studies by Pal et al. demonstrated that OH^- at O5 had no direct-H-bond partner in their S_0 model (see Figure S7a in Ref. [8]). In contrast, they have also reported that they were able to generate an O5–O_{W2} H-bond using "a small model of the oxidized S_0' model" (not "the QM/MM S_0 model", see Figure S7b in Ref. [8]). However, the details of the small, oxidized S_0' model were not provided and the arguments for the presence of the O5–O_{W2} H-bond seem weak.

Our QM/MM calculations resulted in O5–O_{W2} distances of 3.04 Å in the presence of H_2O at W2 and 2.67 Å in the presence of OH^- at W2 (Figure 7). The O5–O_{W2} distance of 3.04 Å is closer to the

distance of 3.1 Å in the PSII crystal structure [9], which indicates that W2 is H_2O rather than OH^- in the crystal structure. Although one may possibly consider that the O5–O_{W2} distance of 2.67 Å is a stronger H-bond, it should also be noted that characteristics of H-bonds cannot be defined by only the distances, as pointed out by Schutz and Warshel [10]. According to Frey [2], an essential requirement for a short H-bond is that the proton lies in-line with the donor and acceptor atoms, whereas the O5–H–O_{W2} angles are 92° (H_2O at W2) and 113° (OH^- at W2) in the QM/MM geometry (Figure 7), far from linearity. We also calculated the δ_H values for OH^- at O5 and found them to be 4.7 (H_2O at W2) and 6.1 (OH^- at W2) ppm (Figure 7), values that are lower than those seen even with weak H-bonds. Thus, it is clear that the hydroxyl O5 has no H-bond partner in the PSII protein environment in the lower S states investigated in the present study, regardless of the W2 protonation state. These results are a further demonstration that characteristics of H-bonds cannot be defined by the distances alone [10].

Proton release from O5 and a water molecule near Cl^- (W446).

One might possibly assume that H_3O^+ can be stabilized at W446 due to the presence of Cl^-. However, W446 preferentially donates H-bonds to both Cl^- and the carbonyl O of D1-Asn181, and it cannot provide an H-bond to W442. Instead, W442 donates an H-bond to W446, which leads to a decrease in the stability of H_3O^+ at W446 and an increase in the barrier for the proton transfer toward the bulk surface (Figure 6c).

Mn oxidation state of the low oxidation state model.

Gatt et al. favored the low oxidation state model, which is two redox equivalents below the high valence model, and proposed that the Mn oxidation state was (III, IV, III, II) for the 2.9-Å structure [11] and (III, III, III, III) for the 1.9-Å structure [12]. They also proposed that a single proton relocation between W2 and D1-His337 triggered the change between (III, III, III, III) and (III, IV, III, II) based on the difference in the Mn_4Ca geometries for the 1.9 Å [9] and 2.9 Å [11] crystal structures [12]. However, recent theoretical studies by Kurashige et al. [13] reported that the Mn oxidation state (III, IV, III, II) is the most relevant state for the 1.9-Å structure [13]. The calculations reported in the present work also find this

valence distribution for the S_{-1} state (Table 1). Thus, it is also possible to have (III, IV, III, II) in the 1.9-Å structure, without altering the protonation state of W2 and D1-His337, and therefore the single proton relocation proposed by Gatt et al [11] is not a unique condition required for switching between the two Mn redox distributions. For further discussions, see Ref. [14] and references therein.

Requirements for long-distance sequential PT. In the O4-water chain, each pair of water molecules is H-bonded with a O_{water}–O_{water} distance of < ~2.8 Å (Table 1). This strongly-coupled chain of water molecules is expected to facilitate the release of protons in a sequential way [15,16]. When O4 is protonated (i.e., pre-PT), the nth H_2O donates an H-bond to the $n^{th}+1$ H_2O along the water chain ("pre-PT" in Supplementary Figure 3). When O4 is deprotonated (i.e., post-PT), the nth H_2O accepts an H-bond from the $n^{th}+1$ H_2O ("post-PT" in Supplementary Figure 3). Because every O atom of the water molecules is linked to another O atom at a distance of < ~2.8 Å along the chain, the water molecules of the chain must undergo an alteration to the entire H-bonding chain as they accept and donate H-bonds, thereby changing the chain from the pre-PT to the post PT pattern, or vice versa. This may explain why the PSII protein environment (i.e., protein dipole preorganization [17]) allows only two H-bond patterns in the O4-water chain: the pre-PT and post-PT patterns (Figure 2).

Further proton transfer pathway toward the bulk surface. In the 1.9-Å structure, the H-bond network of the water chain is terminated at W399. The region of W545, W1047, and W399 may serve as a proton reservoir for a proton released from Mn_4CaO_5 but, further PT toward the lumenal bulk surface may occur. Specifically PT from W399 to PsbU-Asp96 via D1-Asn338 and D2-Asn350 is possible. W399 at the terminus of the in-line water chain is close to the side chain carbonyl O atom of D1-Asn338 (2.68 Å), which is 3.57 Å away from the side-chain N atom of D2-Asn350. The distance of 3.57 Å is longer than a typical H-bond interaction, but not too long for a weak H-bond. Because the side chain N atom of D2-Asn350 is involved in an H-bond network proceeding toward PsbU-Asp96 via W412 (2.80 Å from D2-Asn350) and W324, the crucial step is a PT via the two Asn side chains, because Asn is not a protonatable residue.

Another possibility might be the involvement of more water molecules that are not seen in the 1.9-Å structure. It is likely that almost all stable water molecules, in particular those involved in H-bond networks are clarified at the resolution of 1.9 Å. On the other hand, it seems likely that water molecules exist that are not seen in the crystal structure irrespective of the resolution simply because they are too mobile. The possible presence of such water molecules may compensate for the slightly long H-bond distance of 3.57 Å between D1-Asn338 and D2-Asn350 and promote the PT.

Mn_4CaO_5 model. The ground state of the Mn_4Ca cluster in the S_1 state is considered to be either a singlet or quintet in EPR studies [18-20]. On the other hand, as considered in previous theoretical studies (e.g., [21,22]), the cluster was considered to be in the S_1 state with ferromagnetically coupled Mn atoms; the total spin $S = 7$ and the resulting Mn oxidation state (Mn1, Mn2, Mn3, M4) = (III, IV, IV, III). The resulting optimized Mn_4CaO_5 geometry appears not to be crucial to the spin configurations "within the same Mn redox distribution", as demonstrated in previous theoretical studies [21,22]. It is of note that the two S_2 conformers suggested in Refs. [23-25] to explain the two spin states seen by EPR [26] have different Mn redox distributions, (III, IV, IV, IV) and (IV, IV, IV, III) . (See Ref. [24] for our S_2 state geometry, which is similar to the model, e.g., by Pantazis et al. [23])

Supplementary Methods

The ^1H NMR chemical shifts (δ_H) were calculated by using the GIAOs method [27] implemented in the Qsite [28] program. The absolute shielding constant of ^1H of tetramethylsilane (TMS) was calculated to be 31.6 ppm on the basis of the atomic coordinates in Ref. [29] and used as the TMS reference for δ_H.

Supplementary References

1 Jeffrey, G. A. *An Introduction to Hydrogen Bonding*, Oxford University Press, Oxford (1997).

2 Frey, P. A. *in Isotope Effects in Chemistry and Biology (Kohen, A. and Limbach, H.-H., Eds.)*, pp 975-993, CRC press, Boca Raton, FL (2006).

3 Frey, P. A. Strong hydrogen bonding in molecules and enzymatic complexes. *Magn. Reson. Chem.* **39**, S190-S198 (2001).

4 Saito, K. & Ishikita, H. H atom positions and nuclear magnetic resonance chemical shifts of short H bonds in photoactive yellow protein. *Biochemistry* **51**, 1171-1177 (2012).

5 Saito, K. & Ishikita, H. Formation of an unusually short hydrogen bond in photoactive yellow protein. *Biochimica et biophysica acta* **1827**, 387-394 (2013).

6 Hibbert, F. & Emsley, J. Hydrogen bonding and chemical reactivity. *Adv. Phys. Org. Chem.* **26**, 255-379 (1990).

7 Limbach, H.-H. *et al.* OHO hydrogen bond geometries and NMR chemical shifts: from equilibrium structures to geometric H/D isotope effects, with applications for water, protonated water, and compressed ice. *Isr J Chem* **49**, 199-216 (2009).

8 Pal, R. *et al.* S-State Model of the Oxygen-Evolving Complex of Photosystem II. *Biochemistry* **52**, 7703-7706 (2013).

9 Umena, Y., Kawakami, K., Shen, J.-R. & Kamiya, N. Crystal structure of oxygen-evolving photosystem II at a resolution of 1.9 Å. *Nature* **473**, 55-60 (2011).

10 Schutz, C. N. & Warshel, A. The low barrier hydrogen bond (LBHB) proposal revisited: the case of the Asp... His pair in serine proteases. *Proteins* **55**, 711-723 (2004).

11 Guskov, A. *et al.* Cyanobacterial photosystem II at 2.9-Å resolution and the role of quinones, lipids, channels and chloride. *Nat. Struct. Mol. Biol.* **16**, 334-342 (2009).

12 Gatt, P., Petrie, S., Stranger, R. & Pace, R. J. Rationalizing the 1.9 A crystal structure of photosystem II--A remarkable Jahn-Teller balancing act induced by a single proton transfer. *Angew Chem Int Ed Engl* **51**, 12025-12028 (2012).

13 Kurashige, Y., Chan, G. K. & Yanai, T. Entangled quantum electronic wavefunctions of the Mn_4CaO_5 cluster in photosystem II. *Nat Chem* **5**, 660-666 (2013).

14 Krewald, V. *et al.* Metal oxidation states in biological water splitting. *Chem. Sci.* **6**, 1676-1695 (2015).

15 Stuchebrukhov, A. A. Mechanisms of proton transfer in proteins: localized charge transfer versus delocalized soliton transfer. *Phys Rev E Stat Nonlin Soft Matter Phys* **79**, 031927 (2009).

16 Freier, E., Wolf, S. & Gerwert, K. Proton transfer via a transient linear water-molecule chain in a membrane protein. *Proc Natl Acad Sci U S A* **108**, 11435-11439 (2011).

17 Warshel, A. Electrostatic origin of the catalytic power of enzymes and the role of preorganized active sites. *The Journal of biological chemistry* **273**, 27035-27038 (1998).

18 Dexheimer, S. L. & Klein, M. P. Detection of a paramagnetic intermediate in the S1 state of the photosynthetic oxygen-evolving complex. *Journal of the American Chemical Society* **114**, 2821-2826 (1992).

19 Yamauchi, T., Mino, H., Matsukawa, T., Kawamori, A. & Ono, T. Parallel polarization electron paramagnetic resonance studies of the S1-state manganese cluster in the photosynthetic oxygen-evolving system. *Biochemistry* **36**, 7520-7526 (1997).

20 Britt, R. D., Peloquin, J. M. & Campbell, K. A. Pulsed and parallel-polarization EPR characterization of the photosystem II oxygen-evolving complex. *Annual review of biophysics and biomolecular structure* **29**, 463-495 (2000).

21 Ames, W. *et al.* Theoretical Evaluation of Structural Models of the S(2) State in the Oxygen Evolving Complex of Photosystem II: Protonation States and Magnetic Interactions. *Journal of the American Chemical Society* **133**, 19743-19757 (2011).

22 Isobe, H. *et al.* Theoretical illumination of water-inserted structures of the CaMn4O5 cluster in the S2 and S3 states of oxygen-evolving complex of photosystem II: full geometry optimizations by B3LYP hybrid density functional. *Dalton Trans* **41**, 13727-13740 (2012).

23 Pantazis, D. A., Ames, W., Cox, N., Lubitz, W. & Neese, F. Two interconvertible structures that explain the spectroscopic properties of the oxygen-evolving complex of photosystem II in the S2 state. *Angew Chem Int Ed Engl* **51**, 9935-9940 (2012).

24 Saito, K. & Ishikita, H. Influence of the Ca^{2+} ion on the Mn_4Ca conformation and the H-bond network arrangement in Photosystem II. *Biochimica et biophysica acta* **1837**, 159-166 (2014).

25 Bovi, D., Narzi, D. & Guidoni, L. The S State of the Oxygen-Evolving Complex of Photosystem II Explored by QM/MM Dynamics: Spin Surfaces and Metastable States Suggest a Reaction Path Towards the S State. *Angew Chem Int Ed Engl* **52**, 11744-11749 (2013).

26 Peloquin, J. M. & Britt, R. D. EPR/ENDOR characterization of the physical and electronic structure of the OEC Mn cluster. *Biochimica et biophysica acta* **1503**, 96-111 (2001).

27 Cao, Y. *et al.* Nuclear-magnetic-resonance shielding constants calculated by pseudospectral methods. *J Chem Phys* **122**, 224116 (2005).

28 QSite, version 5.8, Schrödinger, LLC, New York, NY, 2012.

29 Wolf, A. K. *et al.* Predicted crystal structures of tetramethylsilane and tetramethylgermane and an experimental low-temperature structure of tetramethylsilane. *Acta Crystallogr B* **66**, 229-236 (2010).

30 Siegbahn, P. E. Water oxidation mechanism in photosystem II, including oxidations, proton release pathways, O-O bond formation and O2 release. *Biochimica et biophysica acta* **1827**, 1003-1019 (2013).
